# Recent advances in palmoplantar pustulosis

**DOI:** 10.12703/r/10-62

**Published:** 2021-07-27

**Authors:** Alexandra Maria Giovanna Brunasso, Cesare Massone

**Affiliations:** 1 Dermatology Unit, Galliera Hospital, Genoa, Italy

**Keywords:** Palmoplantar pustulosis, psoriasis, pustular psoriasis, therapy

## Abstract

Palmoplantar pustulosis (PPP) is a chronic inflammatory condition where crops of sterile pustules with erythematous keratotic lesions causing bleeding and pain appear on the palms and soles. Recently, the European Rare and Severe Expert Network considered PPP as a variant of pustular psoriasis with or without psoriasis vulgaris.

The prevalence of PPP varies from 0.050 to 0.12%. PPP occurs more frequently in women and the highest prevalence occurred between the ages of 50 and 69 years. Nail psoriasis seems to be frequent in PPP, ranging from 30 to 76%, and psoriatic arthritis in 8.6 to 26% of PPP patients.

Synovitis, acne, pustulosis, hyperostosis, osteitis (SAPHO) syndrome and pustulotic arthro-osteitis are considered PPP-associated disorders. PPP has been reported with other co-morbidities such as psychiatric disorders, thyroid-associated disease, altered calcium homeostasis, gluten sensitivity diabetes, obesity, and dyslipidemia, but larger studies are required to prove such associations. Environmental exacerbating factors might contribute to the onset or worsening of PPP such as cigarette smoking, stress, focal infections, metal allergies, and drug intake. Genetic predisposition plays an important role in PPP.

In PPP, both the innate and the adaptive immune systems are activated. The acrosyringeal expression of IL-17 has been demonstrated, indicating that the eccrine sweat gland is an active component of the skin barrier and an immune-competent structure. Increased levels of several inflammatory molecules, including IL-8, IL-1α, IL-1β, IL-17A, IL-17C, IL-17D, IL-17F, IL-22, IL-23A, and IL-23 receptor, have been detected in PPP biopsies. Increased serum levels of TNF-α, IL-17, IL-22, and IFN-γ have been detected in patients with PPP in comparison to healthy subjects, suggesting a similar inflammatory pattern to psoriasis vulgaris. Oral and tonsillar infections serve as trigger factors for PPP.

Long-term therapy is required for many patients, but high-quality data are limited, contributing to uncertainty about the ideal approach to treatment.

## Definition and classification

**Figure 1.**
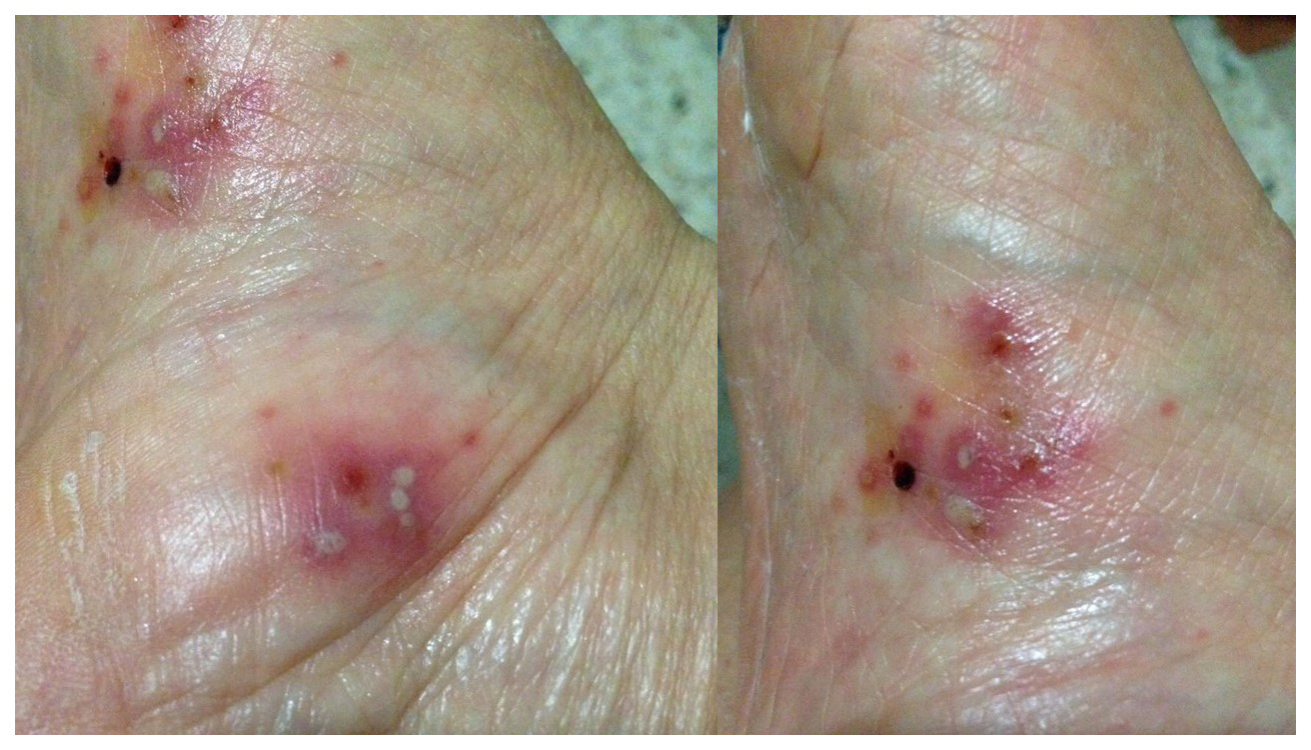
Crops of sterile pustules on the right sole. This image was taken in CM’s clinic for the purpose of this manuscript.

**Figure 2.**
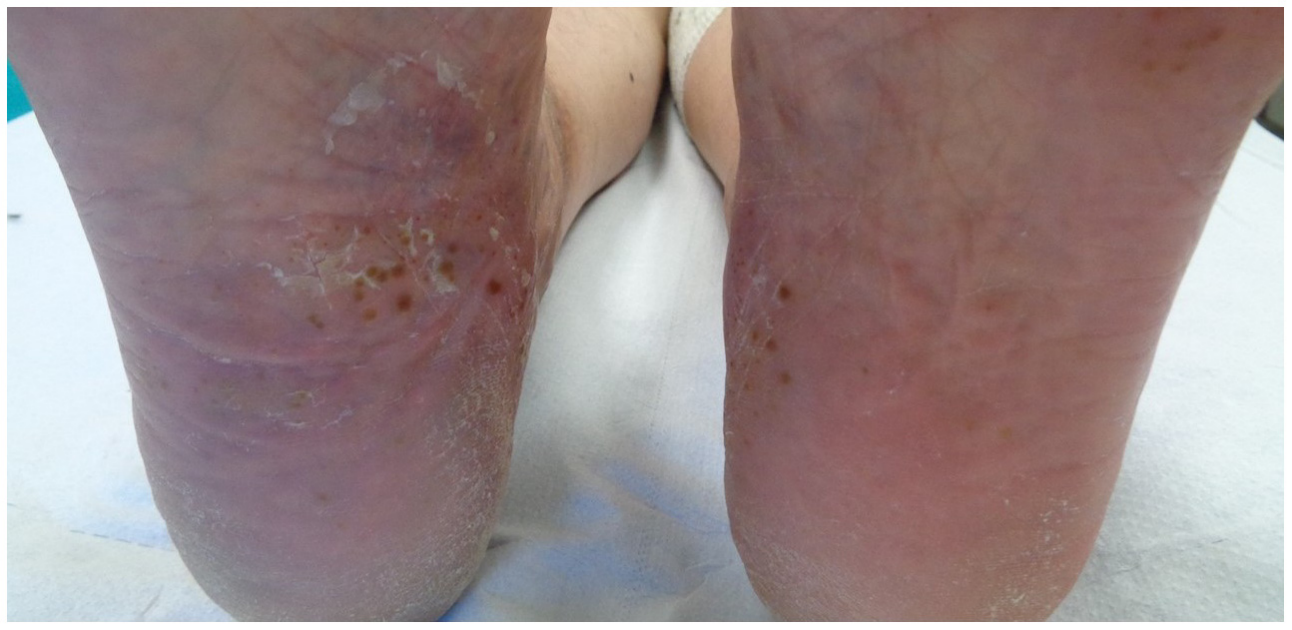
Crops of sterile pustules on both soles. This image was taken in CM’s clinic for the purpose of this manuscript.

Palmoplantar pustulosis (PPP) is a chronic inflammatory condition characterized by the recurrent appearance of crops of sterile pustules on the palms and soles (Figure 1 and Figure 2), in conjunction with erythematous keratotic lesions that tend to crack, causing bleeding and pain^1–4^.

In 1930, Barber assigned this condition the name PPP and categorized it as a local pustular form of psoriasis, but this entity’s etiology and its links to other pathologies are still in contention^1,2,4,5^. Some proposed that PPP is a separate entity, despite PPP and PV sharing particular phenotypes, and in 2007 the International Psoriasis Council separated PPP and psoriasis into distinct entities^1,2,4–6^. In 2017, the European Rare and Severe Expert Network (ERASPEN) distinguished between psoriasis vulgaris (PV) and pustular psoriasis (PP) and recognized them as distinct phenotypes^1,2,4–6^. PPP was considered a variant of PP with the subclassification with or without PV^7^. Such classification of PPP as a form of PP is not widely accepted, mainly in Japan where PPP is considered as a separate disease from palmoplantar PP (PPPP)^8^.

## Epidemiology

Epidemiologic data regarding PPP are limited. The prevalence of PPP across studies varies from 0.050% in Sweden, 0.051% in Korea, and 0.091% in Germany to 0.12% in Japan^1,9^.

PPP occurs more frequently in women than in men; observational studies from across the world have found proportions of female patients ranging from 58 to 94%^1,2^. The age of onset of PPP is usually between the fourth and the fifth decades; the mean age of onset in a retrospective study that we conducted including 39 patients was 48 years^2^. In a nationwide Japanese study published in 2015, the highest prevalence for PPP occurred between the ages of 50 and 69 years^9^.

The prevalence of PV among PPP patients ranges from 14 to 61%^10–13^. Nail involvement (nail psoriasis) in PPP seems to be frequent, ranging from 30 to 76%^2^. A recent study conducted in Seoul evaluated nail involvement in PPP, observing a high prevalence (66.3%) and a correlation between nail changes and severity of the disease, and the predominant findings were onycholysis and crumbling^14^. In Northern European populations, nail involvement has been reported in one-third of cases, with subungual pustulation^13^.

The prevalence of psoriatic arthritis (PsA) in PPP patients has been reported to be from 8.6 to 26%^1,2,13,14^.

## Clinical presentation and severity assessment

PPP’s main clinical characteristic consists of sterile pustules from 1 to 10 mm located on the palms and/or soles with bilateral involvement that might be asymmetrical^1,2^. The pustules usually arise in an erythematous base associated or not with crusting, desquamation, fissures, and/or hyperkeratosis^1,2^. The pustules tend to coalesce, resolving after several days with brown macules and/or the formation of hyperkeratotic plaques resembling plaque psoriasis^1,2^. The involvement of both palms and soles seems to be frequent; the thenar or hypothenar eminences and the central palm are common sites of involvement^1,2,6^. The instep, the medial and lateral borders of the foot across from the instep, and the sides or back of the heel are common sites on the feet^1,2,6^. Diffuse involvement of the whole palmoplantar area is seen in more severe cases^1,2,6^. PPP is characterized by a recurrent course, the severity does not appear to attenuate over time, and the risk of relapse remains during the patient’s lifetime^15,16^.

The impairment of quality of life (QoL) can be significant owing to symptoms such as burning, pain, itch, bleeding of fissures, and complications such as subcutaneous skin infections (erysipelas and cellulitis) that might require hospital admission^1,2^. Severe eruptions might impair the capacity to work, walk, or exercise^1^. In 2017, Trattner *et al*. in a cross-sectional study demonstrated a direct correlation between severity, measured by PalmoPlantar Pustulosis Severity Index (PPPASI), and QoL impairment, measured by Dermatology Life Quality Index (DLQI), data recently confirmed in a larger study including UK and Northern European patients^10,16^. PPP severity seems to be higher in female versus male patients and in current or past smokers^10^.

## Histology

PPP is characterized by the presence of pustules filled with neutrophils and eosinophils in the upper epidermis, psoriasiform epidermal hyperplasia and parakeratosis, and loss of the granular layer^1,17–20^. Spongiosis and epidermal vesicle formation is usually seen in the early stages of PPP^1^. A mixed perivascular and diffuse infiltrate of neutrophils, lymphocytes, eosinophils, and mast cells is present in the dermis with accumulation of eosinophils and mast cells bellow the pustule^1,19,20^. In 2019, Masuda-Kuroki *et al*., comparing the histologic presentations of PPP and pompholyx, found two main key features that differentiated both diseases: the presence of vesicles with spongiosis in pompholyx and microabscesses on the edges of vesicles in PPP^17^. Hyaluronate accumulation in the epidermis and presence of acantholytic keratinocytes covered with hyaluronate are additional features typical of pompholyx and absent in PPP^18^.

## PPP-associated disorders

Synovitis, acne, pustulosis, hyperostosis, osteitis (SAPHO) syndrome is a very rare condition that is more frequent in Asia and characterized by the presence of synovitis, acne, pustulosis, hyperostosis, and osteitis^1^. It may present with PPP or other neutrophilic diseases (acne conglobata, acne fulminans, hidradenitis suppurativa, dissecting cellulitis of the scalp, and PP) and is associated with anterior chest wall arthropathy^1^.

Pustulotic arthro-osteitis (PAO), involving the anterior chest wall, is frequently (20–30%) reported in Japanese patients with PPP; focal infections are a known trigger factor^1,21^. PAO is more prevalent than SAPHO syndrome, and both diseases are characterized by increased uptake in bone scintigraphy of the anterior chest wall^21^.

## PPP comorbidities

The relationship between PPP and systemic diseases is controversial^1^. Psychiatric disorders (depression, anxiety, schizophrenia, and manic-depressive disease) have been reported with a high incidence among female PPP patients (23–24%) compared to healthy controls (5%)^22^. Some authors have reported hypertension, thyroid-associated disease, altered calcium homeostasis, diabetes, obesity, and dyslipidemia, but larger studies are required to prove such associations^16,22^. Increased prevalence of gluten sensitivity in PPP patients was reported in a Swedish study, but these findings were not confirmed in the German and Austrian populations, suggesting geographical or ethnic variability^16^.

## Trigger factors

Several studies have suggested that environmental exacerbating factors might contribute to the onset or worsening of PPP such as cigarette smoking, stress, focal infections, metal allergies, and drug intake^1,22^.

### Tobacco smoke

The high prevalence of current or past smoking in PPP patients (42 to 100%) suggests a role in the pathogenesis of the disease; smoking withdrawal seems to improve the severity of PPP^23^. Nicotine has been suggested to exert a role on the sweat glands or on the keratinocytes, increasing cornification and promoting neutrophilic inflammation^1,22^. Differences in nicotine acetylcholine receptor staining patterns in PPP skin have revealed an abnormal response to nicotine that might contribute to inflammation^1,22^. Recently, Kobayashi *et al*. demonstrated a direct relationship between the severity of PPP (measured by PPPASI) and smoking index in female PPP patients. Moreover, they demonstrated that there was a synergistic release of IL-17A and IL-36γ after exposure to cigarette smoke extract; IL-36γ was highly expressed in tonsillar epithelial cells of PPP patients in comparison with patients without PPP, suggesting that IL-36γ production might occur in tonsil tissues as a result of a smoking habit^24^.

### Focal infections and microbiome

Acute or chronic infections such as sinusitis, dental infection, and tonsilitis have been identified as trigger factors for PPP^1^. In 2017, Kuono *et al*. demonstrated that oral focal infections are directly related to PPP severity and the control of such infections with the use of tonsillectomy improves disease severity^25^. Recently, a higher incidence of oral dysbiosis in PPP patients has been demonstrated as compared to healthy subjects^26,27^. In addition, Kuono *et al*. suggested that oral dysbiosis in Japanese patients might play a role in both PPP and PAO^4^. Several authors have described the presence of microbiome inside the pustules in 30% of cases, in particular *Pseudonocardia, Devosia*, and *Staphylococcus*, which are the most frequent species among smokers, and recently also of *Malassezia* spp.^3,28^. Takahara *et al*. in 2018 demonstrated the positive role of tonsillectomy in improving PPP lesions^29^.

### Stress

PPP patients report worsening or new onset of lesions during stressful times, and high levels of anxiety have been reported in such patients^1^. An increased number of contacts between nerves and mast cells and intense substance P-like immunoreactivity in neutrophils in pustules and the papillary dermis of PPP patients suggest a role for neuro-mediation in the inflammatory process^1^.

### Drug intake

Paradoxical adverse events (PAEs) associated with biological therapy, mainly during anti-TNF-α therapy, have been reported in 2–5% of patients; PPP is the most common skin manifestation of PAE^30,31^. The new onset of PPP in such patients represents an ongoing type-I IFN-driven innate immune response in the absence of T cell-driven inflammation^30^. Interestingly, type-I IFN is the main inflammatory pathway involved in acute and unstable psoriasis forms such as guttate, erythrodermic psoriasis, and PPP induced by anti-TNF-α therapies^32^.

### Metal allergies

In the literature, there are different isolated reports of the association of PPP and allergies. In a systematic review covering 519 PPP patients, the frequency of allergies among PPP patients was 22.7%; among the identified allergens, 84.3% of cases corresponded to metal allergies^33^. The exacerbation of PPP lesions after patch-test (effect only described with metals and not with other allergens) and the improvement of PPP in 65% of cases after withdrawal from metal contact support these findings^33^.

## Genetic background

PPP has been considered a separate disease from PV by the International Psoriasis Council based on genetic differences, mainly the fact that PPP is not associated with the most common PV locus, PSORS1, but it is worth noting that many other genetic links between the two diseases have been found^1^. Variations of *IL19*, *IL20*, and *IL24* genes have been reported as risks factors for both diseases^1,34–36^. In addition, missense variants in the *CARD14* gene have been detected in both PPP as well as PV and generalized PP (GPP)^34,35^. Recently, mutations in AP1S3 have been described in PPP patients, representing another overlap of genetic risk factor among psoriasis, PPP, and GPP^1,34–36^. The ATG16L1 mutation in a gene located on chromosome 2 that plays a role in the immunological response of psoriasis, by affecting the production of antimicrobial peptides and the production of IL-18 and IL-1, leads to a propagating systemic inflammation that has been detected in both PPP and PV (with single nucleotide polymorphisms)^1,34–37^. IL36RN loss-of-function mutation induces the activation of IL-36 signaling, causing unregulated cytokine release and skin inflammation manifested mainly as GPP^37^. The frequency of IL36RN mutations among GPP patients is high (75–80%); such mutations have also been described in acrodermatitis continua of Hallopeau (ACH), in inverse psoriasis associated with PP, and in PPP with a lower frequency, suggesting a common genetic predisposition for all forms of PP, some of them associated with PV^38^. Such a link between PP and PV is supported by the genetic findings of PPP patients with and without concomitant plaque psoriasis; gene expression microarray studies of skin biopsies from PPP with concomitant plaque psoriasis and skin biopsies from PPP without concomitant plaque psoriasis have revealed that PPP with or without concomitant plaque psoriasis cannot be distinguished on the basis of gene expression, which suggests a relationship between these conditions^1,34^. Increased expression of the *IL17* gene without an increase in IL-23 gene expression has been found in PPP, both with and without concomitant plaque psoriasis^1,34^.

## Pathogenesis

In PPP, both the innate and the adaptive immune systems play a role, in addition to environmental factors and genetic susceptibility^1^. The intraepidermal sweat duct (acrosyringium) appears to be the primary site of inflammation, where vesicle/pustule formation takes place in PPP^1,39^. The acrosyngeal expression of IL-17 has been demonstrated, indicating that the eccrine sweat gland is an active component of the skin barrier and an immune-competent structure^40^.

Abnormalities of eccrine sweat gland function in the palmoplantar region might contribute to the formation of vesicles and pustules in PPP^1^. Increased access to medical care for PPP patients in the summer season compared with the cold season suggests a role for sweating as a trigger for PPP^1^. Increased numbers of Langerhans cells around the eccrine sweat ducts point towards an antigen-driven process associated with infiltration of lymphocytes, neutrophils, eosinophils, and mast cells that destroys the acrosyringium. Increased levels of several inflammatory molecules, including IL-8 (neutrophil chemoattractant), IL-1α, IL-1β, IL-17A, IL-17C, IL-17D, IL-17F, IL-22, IL-23A, and IL-23 receptor, have been detected in PPP biopsies^1^. Increased serum levels of TNF-α, IL-17, IL-22, and IFN-γ have been detected in patients with PPP in comparison to healthy subjects, suggesting a similar inflammatory pattern to PV^1,39–43^. In the acrosyringium, there is overexpression of IL-17, IL-8, and IL-36γ^1,39–43^. Such expression is induced by antimicrobial peptides as LL-37 or TLN-58, suggesting that targeting IL-17 might not be sufficient to control the disease^1,39–43^. Oral and tonsillar infections serve as trigger factors for PPP^1,44^. A hyperimmune response to *streptococci* has been demonstrated in tonsillar mononuclear cells of PPP patients, suggesting that tonsillar-induced autoimmune/inflammatory responses might play a role in PPP pathogenesis^44^. Cutaneous lymphocyte antigen (CLA), chemokine receptor CCR6, and beta1-integrin serve in the recruitment of T cells from the tonsils to the skin^44^.

Innate immunity is upregulated in PPP lesions, as demonstrated by the high expression of IL-1α, IL-1β, IL-8, and IL-36γ. IL-1β is the main inducer of lipocalin 2 (LCN2) production by keratinocytes. LCN2 is a potential blood biomarker candidate for PPP, capable of chemoattraction of neutrophils, seen not only in PPP but also in hidradenitis suppurativa^45^. IL-8 is overexpressed in PPP skin biopsies compared to PV and healthy skin; this finding has been proved in a clinical setting using an anti-IL-8 monoclonal antibody that obtained a good clinical response in PPP but has failed to obtain efficacy in a phase II trial in PV^43^.

IL-36γ levels have been found to be increased in PPP and PV lesions compared to healthy skin; positive staining can be seen in the sweat duct cells in the dermis and in keratinocytes nearby PPP pustules, suggesting a role in pustule formation. IL-36γ promotes IL-8 secretion by keratinocytes, which leads to chemoattraction of neutrophils and results in pustule formation^41,43^.

## Personal view

In the last 10 years, it has been proposed that the onset of aseptic pustular lesions in the palms and soles be classified into two different conditions: PPP and PPPP. Both entities share the same clinical presentation on the palmoplantar area and identical nail features, concomitant PsA may be present in both, and the histopathologic findings are quite similar. The key differentiating points are the presence of PV in other sites and the higher frequency of a family history of psoriasis in the PPPP variant. PPP has been recently classified as a form of PP, and it is worth noting that GPP and ACH are also forms of PP that could be associated with PV or not, but only in the case of PPP has a separate entity—such as PPPP—been proposed. From a genetic point of view, PV and PP share some common genetic susceptibility links and certainly differ by the higher prevalence of PSORS1 in PV. Regarding therapy, PPP seems to be more difficult to treat than PV, but such a lower response to both conventional and biological therapies has also been seen on palmoplantar plaque psoriasis^46^.

Considering all the previously cited factors, we consider the term PPP with or without PV enough to describe the condition; furthermore, nomenclature uniformity allows a better recognition of the disease.

## New therapeutic options

As PPP is a chronic and recurrent disease with a high impact on QoL, long-term therapy is required for many patients, but high-quality data are limited (small sample sizes in randomized controlled trials [RCTs], geographical variability), contributing to uncertainty about the ideal approach to treatment^47^. Recently, new biological and non-biological molecules have been investigated in PPP patients^47,48^.

Several case reports have recorded the efficacy of TNF-α-blocking agents in PPP despite the paradoxical onset during such therapies^46,48^. One 24-week clinical trial supports the role of etanercept for PPP, and further evidence of efficacy in several cases affected by SAPHO syndrome have been reported under different TNF-α-blocking agents (infliximab, adalimumab, and golimumab)^49^.

A 16-week RCT failed to achieve efficacy in PPP patients treated with ustekinumab (45 mg given at weeks 0 and 4) versus placebo, but several reports (case series and one uncontrolled study) using a higher dose (90 mg) of ustekinumab demonstrated efficacy in PPP patients^50–53^.

Anti-IL-17 monoclonal antibodies have been tested on PPP. The 2PRECISE trial evaluated secukinumab at 300 mg or 150 mg versus placebo given at weeks 0, 1, 2, 3, and 4 and then every 4 weeks^54^. The trial did not achieve the primary endpoint of superiority of secukinumab (300 or 150 mg doses) over placebo at week 16 (PPPASI-75 achieved by 27%, 18%, and 14%, respectively)^54^. But, in the long-term, at week 52, non-responders of the secukinumab and the placebo arms were treated with 300 mg every 4 weeks with 42% PPPASI-75 efficacy and 150 mg with 35% PPPASI-75 efficacy^54^. Only case reports regarding other anti-IL-17 therapies are available, with lack of efficacy of brodalumab in four PPP cases, but a phase III RCT is ongoing^43,55^.

Anti-IL-23 therapies seem to be promising for PPP patients. Two phase III RCTs demonstrated the efficacy of guselkumab versus placebo in PPP^56,57^. In the guselkumab 100 mg group, 57% of PPP patients had at least 50% improvement in PPPASI total score (PPPASI-50) at week 16 compared to 34% in the placebo group^56,57^. At week 52, PPPASI-75 was reached in 55.6% of patients treated with 100 mg and in 59.6% of patients treated with 200 mg of guselkumab^56,57^.

The systemic retinoid alitretinoin has been evaluated in a few PPP patients, demonstrating a 45.2% improvement at week 24, but this result was not significantly better than placebo; additional studies are required^58^. Few cases reports and two retrospective studies have reported the efficacy of apremilast in PPP both in monotherapy and in association with other systemic therapies, but RCTs are missing^59,60^. Until today, there are no reports regarding the use of ixekizumab, risankizumab, or tildrakizumab in PPP. Improvement in PPP and ACH has been recorded during treatment with anakinra in a few case reports^43,47^. Canakinumab has been found to be ineffective for PPP in one report^43,47^. The APRICOT trial is currently evaluating anakinra versus placebo for PP, but the results are still awaited^61^.

New molecules under development for PPP include an anti-IL-8 monoclonal antibody (HuMab 10F8) that in an open-label trial has demonstrated a decrease of 50% or more in new pustule formation at week 8 in 67% of PPP patients^43^. In addition, an oral formulation that blocks CXCR2 (IL-8B receptor), RIST4721, is currently under investigation in a phase IIa RCT^43^.

Blocking the IL-36 pathway has recently been under the spotlight for PP, and for GPP in particular^62^. One phase IIb dose-finding RCT is currently evaluating the PPPASI response at week 16 for spesolimab in PPP as the primary endpoint, and a phase IIa RCT of spesolimab versus placebo is currently ongoing, with PPPASI-50 at week 16 as the primary outcome^43^. We need to wait for the results of such studies to understand the potential role of anti-IL-36 therapies in PPP.
